# Targeting PDGF/PDGFR Signaling Pathway by microRNA, lncRNA, and circRNA for Therapy of Vascular Diseases: A Narrow Review

**DOI:** 10.3390/biom14111446

**Published:** 2024-11-14

**Authors:** Chao-Nan Ma, Shan-Rui Shi, Xue-Ying Zhang, Guo-Song Xin, Xiang Zou, Wen-Lan Li, Shou-Dong Guo

**Affiliations:** 1 Institute of Lipid Metabolism and Atherosclerosis, School of Pharmacy, Shandong Second Medical University, Weifang 261053, China; machaonan21@aliyun.com (C.-N.M.); shishanrui0316@aliyun.com (S.-R.S.); zhangxueying3@aliyun.com (X.-Y.Z.); 2 School of Pharmacy, Engineering Research Center for Medicine, Harbin University of Commerce, Harbin 150076, China; 103009@hrbcu.edu.cn (G.-S.X.); zouxiang@hrbcu.edu.cn (X.Z.)

**Keywords:** cardiovascular disease, non-coding RNA, platelet-derived growth factor, respiratory disease, vascular system

## Abstract

Despite the significant progress in diagnostic and therapeutic strategies, vascular diseases, such as cardiovascular diseases (CVDs) and respiratory diseases, still cannot be successfully eliminated. Vascular cells play a key role in maintaining vascular homeostasis. Notably, a variety of cells produce and secrete platelet-derived growth factors (PDGFs), which promote mitosis and induce the division, proliferation, and migration of vascular cells including vascular smooth muscle cells (SMCs), aortic SMCs, endothelial cells, and airway SMCs. Therefore, PDGF/PDGR receptor signaling pathways play vital roles in regulating the homeostasis of blood vessels and the onset and development of CVDs, such as atherosclerosis, and respiratory diseases including asthma and pulmonary arterial hypertension. Recently, accumulating evidence has demonstrated that microRNA, long-chain non-coding RNA, and circular RNA are involved in the regulation of PDGF/PDGFR signaling pathways through competitive interactions with target mRNAs, contributing to the occurrence and development of the above-mentioned diseases. These novel findings are useful for laboratory research and clinical studies. The aim of this article is to conclude the recent progresses in this field, particular the mechanisms of action of these non-coding RNAs in regulating vascular remodeling, providing potential strategies for the diagnosis, prevention, and treatment of vascular-dysfunction-related diseases, particularly CVDs and respiratory diseases.

## 1. Introduction

Vascular cells play vital roles in both physiological and pathological conditions. Notably, smooth muscle cells (SMCs) are the primary cell type in the media of the blood vessel wall, and these cells are involved in vascular remodeling and maintaining vessel homeostasis. Emerging evidence have demonstrated that vascular SMCs (VSMCs) are highly plastic. Upon vascular injury or the stimulation of cytokines and growth factors, VSMCs may switch from a “contractile” phenotype to a “synthetic” phenotype. This transition is characterized by the increased proliferation, decreased contractility, and secretion of some degrading enzymes, thereby affecting vascular remodeling [1,2]. Moreover, vascular endothelial cells are the first to bear the brunt of vascular risk factors; these cells are closely related to vascular health. It has been suggested that endothelial function can be used to monitor the response to lifestyle changes and the effects of drug interventions, thereby providing valuable information for vascular risk factors and identifying effective treatment strategies for patients [3]. Collectively, vascular cells determine the final outcome of many cardiovascular diseases (CVDs) including restenosis, atherosclerosis, and aortic aneurism, and respiratory diseases, such as pulmonary arterial hypertension (PAH) and asthma [2]. Given that vascular diseases have a high morbidity and mortality globally and are considered to be a serious public health problem [4], searching for novel therapeutic strategies is still a research hotspot and long-lasting challenge in this field.

Platelet-derived growth factors (PDGFs) are discovered more than two decades ago. They are composed of five different disulphide-linked dimers including PDGF-AA, PDGF-BB, PDGF-AB, PDGF-CC, and PDGF-DD, that are built up of four different gene-encoded polypeptide chains including PDGF-A, PDGF-B, PDGF-C, and PDGF-D. These five dimers exert their function through two receptor tyrosine kinases, PDGF receptor (PDGFR) alpha/α and beta/β [5,6]. Interestingly, PDGF-A and PDGF-B are intracellular-activated during transport in the exocytic pathway for subsequent secretion, while PDGF-C and PDGF-D are secreted as latent factors and activated by extracellular proteases [5]. Notably, PDGFs are critical regulators of animal development and homeostasis; generally, they promote cell proliferation, survival, and migration. Dysfunction of the PDGF signaling pathway has been observed in a wide array of pathological conditions, such as cancer, respiratory diseases, and CVDs. The abnormalities of the PDGF signaling pathway include the amplification of PDGFRs, the gain of function mutations, and the activation of chromosomal translocations [7]. Notably, accumulating evidence has demonstrated that PDGF-BB is an important inducer of vascular cell pathogenesis because it can bind with all isoforms of PDGFRs [6,8], thereby activating various signaling pathways that are involved in the pathogenic transmission of vascular cells [9].

The potential of targeting the PDGF/PDGFR signaling pathway for cancer therapy has been reviewed in the year of 2022 [6]. Given PDGFs have been demonstrated to regulate vascular cells in recent years, and, particularly, microRNA (miRNA), long non-coding RNA (lncRNA), and circular RNA (circRNA) are involved in the regulation of PDGF/PDGFR signaling pathways through competitive interactions, it is interesting to conclude the recent findings in this field, providing potential mechanisms of action of vascular remodeling and therapeutic strategies for the treatment of vascular-dysfunction-related diseases, particularly CVDs and respiratory diseases. In this review, the related literatures were obtained from the database, mainly including PubMed and Web of Science using “PDGF or PDGFR” in combination with “microRNA, lncRNA and circRNA” as keywords.

## 2. A Brief Review of PDGF/PDGFR Signaling Pathways in Vascular Cells

**Figure 1:**
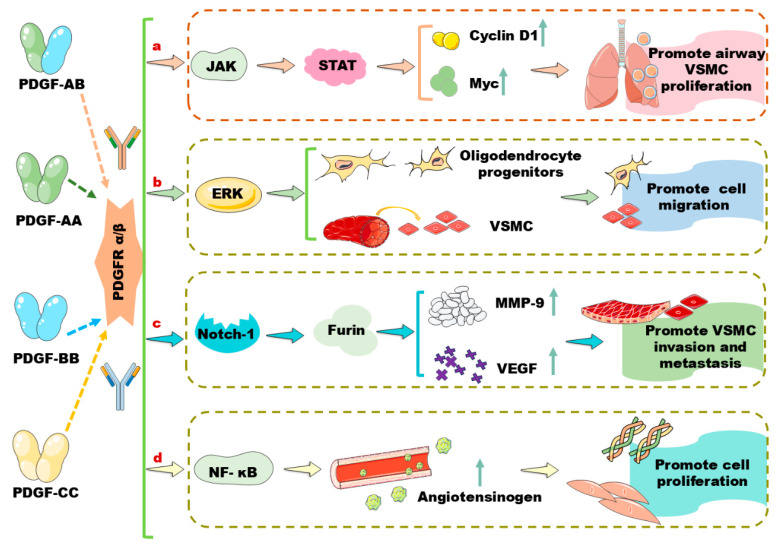
PDGF dimers bind PDGFRα and PDGFRβ to activate distinct signaling pathways that are involved in the regulation of vascular cell proliferation, migration, and invasion. According to the available references, four different disulphide-linked dimers including PDGF-AA, PDGF-AB, PDGF-BB, and PDGF-CC are involved in regulating the phenotype switch of vascular cells, thereby regulating vessel homeostasis in different organs. ERK: extracellular signal-regulated kinase; JAK: Janus kinase; MMP: matrix metalloproteinase; Myc: myelocytomatosis oncogene gene; NF-κB: nuclear factor kappa-B; PDGF: platelet-derived growth factors; PDGFR: PDGF receptor; STAT: signal transducer and activator of transcription; VEGF: vascular endothelial growth factor; VSMC: vascular smooth muscle cells.

PDGF-A and PDGF-B are expressed in many cell types. In VSMCs, PDGFs stimulate the expression of the tyrosine phosphorylation of phospholipase C, inducing cell proliferation and migration [10]. PDGF-AB can combine with PDGFRα to activate the signal transducer and activator of transcription (STAT) signaling pathway through the action of the Janus kinase (JAK) and sarcoma enzyme, and upregulate the expression of cyclin D1 and Myc to promote cell proliferation in human airway VSMCs (Figure 1a) [11]. In circulation, shear stress stimulates the secretion of PDGF-BB, which activates SMC chemotaxis and migration [12]. It has been demonstrated that PDGF-AA and PDGF-BB induce the migration of oligodendrocyte progenitors and the chemotaxis and migration of VSMCs, respectively, via activating PDGFRα and the subsequent extracellular signal-regulated kinase (ERK) signaling pathway (Figure 1b) [13,14]. PDGF-BB also induces the proliferation of primary human mesangial cells via activating the mitogen-activated protein kinase (MAPK)/ERK1/2 signaling pathway through PDGFRβ [15]. Moreover, preosteoclast-secreted PDGF-BB induces the differentiation of endothelial progenitor cells into mature endothelial cells in vitro [16]. In endothelial cells, PDGF-BB activates Notch-1 and induces Notch-1/Furin interaction, and upregulates the expression of matrix metalloproteinase (MMP) 9 and vascular endothelial growth factor (VEGF), stimulating cell invasion and metastasis (Figure 1c) [17,18]. Moreover, the activated Notch signaling pathway further upregulates the expression of PDGFRβ, promoting VSMC differentiation [19]. It is known that PDGF-C is expressed predominantly in VSMCs and PDGF-D is expressed primarily in fibroblastic adventitial cells. These two monomeric polypeptide chains can transduce proliferation and migration signals to pericytes and SMCs [20]. For instance, neonatal rat-derived aortic SMCs secrete PDGF-C to promote SMC proliferation in response to vascular injury [21]. Mechanistically, PDGF-C promotes the proliferation of VSMCs and fibroblasts in renal fibrosis via acting on nuclear factor kappa-B (NF-κB) and the Ras-induced upregulation of angiotensinogen (Figure 1d) [22]. PDGF-CC, secreted by alternatively activated macrophages, induces the expression of α-smooth muscle actin in both dermal and gingival fibroblasts and reduces the differentiation of myofibroblast, contributing to fibrosis [23,24]. Moreover, the inhibition of VEGF-A increases the expression of PDGF-CC and its receptors including PDGFRα and PDGFRβ, inhibiting pathological angiogenesis [25]. The PDGF-induced primary actions in vascular cells are concluded in Figure 1. In the following section, we will discuss the roles of miRNA, lncRNA, and circRNA in the regulation of the PDGF/PDGFR signaling pathway in vascular cells and their therapeutic potentials for the diagnosis and treatment of CVDs and respiratory diseases.

## 3. miRNA in Regulation of PDGF/PDGFR Signaling Pathway in Vascular Cells

miRNAs are a class of endogenous 18–24-nucleotide non-coding RNAs that negatively regulate gene expression [1,26]. Accumulating evidence has demonstrated that miRNAs control the expression of multiple genes that are involved in vascular cells including VSMCs, aortic SMCs, airway SMCs, and endothelial and pulmonary artery smooth muscle cells (PASMCs). For example, miRNAs regulate the proliferation, differentiation, and migration of blood vessels by acting on specific signaling pathways including phosphatidylinositol 3-kinase (PI3K), NF-κB, and transforming growth factor-β1 (TGF-β1), thereby modulating inflammation. In addition, miRNAs have an impact on the vital activities of leukocytes, regulating the infiltration and adhesion of leukocytes, effectively modulating inflammation and cell dynamics. miRNAs also regulate protein kinases, cardiac muscle proteins, and various cell growth factors, which target vascular cells and inhibit vascular proliferation and vascular lesions. Notably, miRNAs are potential therapeutic targets to prevent vascular lumen constriction during atherosclerosis and restenosis and modulate lipid metabolism, posing important regulatory roles in the pathogenesis of CVD. Collectively, these miRNAs provide multiple potentials for the treatment of vascular diseases including atherosclerosis, stent restenosis, coronary artery disease (CAD), and heart failure, as well as respiratory diseases, such as PAH and asthma [27,28]. Importantly, accumulating evidence has demonstrated that miRNAs are involved in the direct or indirect regulation of PDGF/PDGFR signaling pathways in the above-mentioned vascular cells. In the following section, we will first summarize the regulatory roles of miRNAs in vascular PDGF/PDGFR signaling pathways as shown in Table 1.

### 3.1. Regulation of PDGF/PDGFR Signaling by miRNAs in VSMC

**Figure 2:**
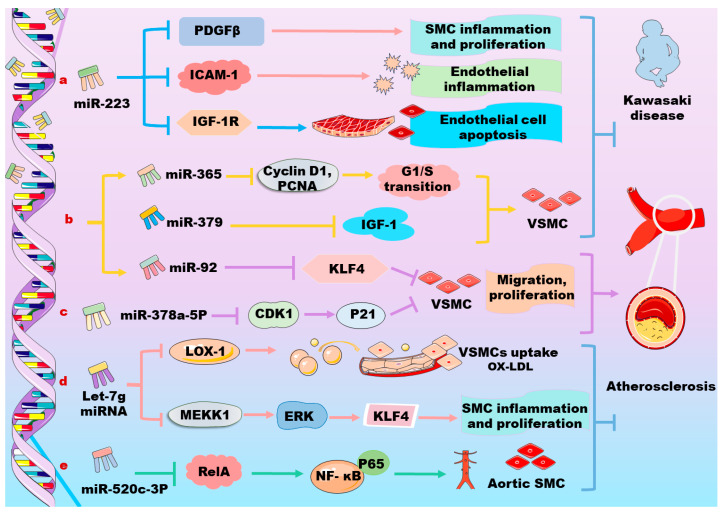
miRNAs target genes that are involved in the PDGF/PDGFR signaling pathways, modulating vascular cell phenotype switch and vascular diseases, such as atherosclerosis and Kawasaki disease. miRNAs are found to regulate various signaling pathways including CDK1/P21, IGF-1, KLF4, CyclinD1, PCNA, NF-κB p65, and MEKK1/ERK/KLF4 in different vascular cells. CDK1: cyclin-dependent kinase 1; ICAM-1: intercellular cell adhesion molecule-1; IGF: insulin-like growth factor; IGF-1R: IGR-1 receptor; KLF4: Krüppel-like factor 4; LDL: low-density lipoprotein; MEKK1: mitogen-activated protein kinase kinase 1; LOX-1: lectin-like oxidized LDL receptor-1; OX-LDL: oxidized low-density lipoprotein; PCNA: proliferating cell nuclear antigen; P21: kinase inhibitor cdkn1a; RelA: reticuloendotheliosis viral oncogene homolog A; SMCs: smooth muscle cells.

PDGFs are potent mitogens for VSMCs. They act on PDGFRs in VSMCs and promote the migration and proliferation of these cells. miRNAs are involved in these processes as shown in Figure 2. For instance, the overexpression of miR125a-5p or miR7 ameliorates the PDGF-BB-induced proliferation, migration, and invasion of VSMCs. miR223 transferred by platelet-derived microparticles (PMPs) or platelets is involved in the functional regulation of vascular cells. Platelet-derived miR223 inhibits VSMC differentiation and restores Kawasaki-disease-induced vascular injury through the inhibition of PDGFRβ; miR143 and miR145 target Krüppel-like factor (KLF) 4 and 5, respectively, to suppress VSMC dedifferentiation (Figure 2a) [29,30]. Platelet deficient in miR223 leads to coronary artery pathology as platelet uptake fails to suppress VSMC dedifferentiation. Thus, targeting the miR223/PDGFRβ axis may provide a novel therapeutic option for Kawasaki-disease-induced vascular pathologies (Figure 2a) [31]. Moreover, PDGF mediates the formation of podosome that is involved in SMC migration through several key regulators including PDGFRα, protein kinase C ε, and P53. Interestingly, miR143 and miR145 can target the regulators of podosome formation, exhibiting therapeutic potential for vascular diseases [32]. miR149-5p (or miR149) targets the proapoptotic factor p53 [33], and affects cell proliferation via negatively regulating JunB [34]. Mothers against decapentaplegic homolog (Smad) 4 is an effective downstream signal transducer of the transforming growth factor (TGF)-β signaling pathway [35]. Notably, miR145-5p directly targets Smad4, thereby suppressing PDGF-induced VSMC proliferation and migration through the downregulation of the TGF-β1/Smad cascade [36]. miR214, a profibrotic miRNA, targets anti-mitogen-inducible gene 6, a negative regulator of epidermal growth factor receptor (EGFR) signaling, thereby promoting TGF-β1-induced profibrotic gene expression [37].

Ying Yang 1 (YY1) inhibits VSMC differentiation and migration by repressing p21WAF1/Clip1 transcription and p21WAF1/Clip-1Cdk4-cyclin D1 assembly [38,39]. Notably, YY1 is negatively regulated by miR29a and miR147b in VSMCs. For instance, the overexpression of miR147b increases the proliferative and migratory abilities of VSMCs, while the knockdown of this miRNA suppresses PDGF-BB-induced cell proliferation and migration possibly via targeting YY1. Furthermore, the overexpression of miR147b increases the Wnt/β-catenin signaling pathway in VSMCs [40]. miR663 plays a key role in the VSMC phenotypic switch upon PDGF stimuli in vitro and in vivo potentially via targeting the transcription factor JunB, a major component of activator protein 1, thereby decreasing the expression of downstream genes including myosin regulatory light chain 9 and MMPs, leading to the inhibition of VSMC migration [41]. miR320 inhibits the proliferation and migration of VSMCs in both basal and PDGF-stimulated conditions potentially by targeting neuropilin 1; this miRNA is useful for an early diagnosis and the treatment of proliferative vascular diseases [42]. Moreover, the overexpression of miR340 promotes VSMC proliferation and invasion potentially via targeting von Hippel–Lindau, a molecule that is involved in multiple physiological processes including angiogenesis, proliferation, and apoptosis [43]. miR1246 is involved in a variety of diseases including myocardial ischemia reperfusion and spinal neuronal disease [44]; this miRNA enhances the proliferation and differentiation of VSMCs via negatively regulating the cystic fibrosis transmembrane conductance regulator [45].

Upon vascular injury, VSMCs switch from a contractile phenotype to a synthetic phenotype, leading to atherogenesis and arterial restenosis. Accumulating evidence has demonstrated that an increased expression of some molecules that are involved in the PDGF-BB signaling pathway is associated with CVDs [46], and the inhibition of PDGF-BB-related pathways has been shown to exert beneficial effects on cardiovascular disorders [47]. Interestingly, miR125a-5p suppresses the growth, migration, and invasion of PDGF-BB-stimulated VSMCs via targeting EGFR mRNA, indicating its potential role for CVD therapy [30]. miR92 expression is significantly upregulated in PDGF-BB-stimulated VSMCs and the mimic of miR92 is demonstrated to enhance VSMC proliferation and migration via targeting Krüppel-like factor 4 (KLF4). Therefore, miR92 is useful for the diagnosis and treatment of vascular restenosis and injury, arteriosclerosis, and other proliferative vascular diseases (Figure 2b) [48]. The expression of miR378a-5p is upregulated in both human atherosclerotic vascular tissues and proliferative VSMCs. Notably, miR378a-5p negatively regulates the expression of cyclin-dependent kinase 1 (CDK1) and its downstream molecule p21 after PDGF-BB stimulation to promote VSMC proliferation, suggesting a novel therapeutic strategy via targeting miR378a-5p in the prevention of PDGF-BB-stimulated atherosclerosis and stent restenosis (Figure 2c) [49]. miR146b-3p is a potential modulator in atherosclerosis development via targeting phosphoinositide-3 kinase catalytic subunit-gamma, thereby inhibiting cell proliferation and migration and reversing the phenotype switch of VSMCs [50].

Several studies have demonstrated that miRNAs may modulate atherosclerosis through the regulation of the NF-κB signaling pathway. For instance, miR520c-3p agomiRNA decreases atherosclerotic plaque size and collagen content potentially via inhibiting the reticuloendotheliosis viral oncogene homolog A (RelA)/p65 NF-κB signaling pathway in VSMCs of the apolipoprotein E-deficient mice [51]. The let-7 miRNA family includes 12 highly conserved isoforms and plays a critical role in modulating inflammatory responses through the regulation of PDGF and NF-κB signaling pathway. For instance, the overexpression of let-7 in VSMCs inhibits inflammatory responses including proliferation, migration, and monocyte adhesion through the regulation of PDGF and tumor necrosis factor (TNF)-α signaling pathways; the depletion of Lin-28 Homolog B, a negative regulator of miRlet-7, leads to the suppression of the TNF-α-induced upregulation of interleukin (IL)-6 and PDGFR in VSMCs; and the restoration of let-7 levels suppresses vascular inflammation mediators including IL-6, IL-1β, and NF-κB, thereby attenuating atherosclerosis as well as diabetes [52]. Moreover, PDGF-B and mitogen-activated protein kinase kinase 1 (MEKK1) are target genes of let-7g. Let-7g can directly suppress the PDGF-B- and PDGF-BB-activated MEKK1/ERK/KLF4 signaling pathway in VSMCs (Figure 2d) [53]. Moreover, miRlet-7g prevents the uptake of oxidized low-density lipoprotein (LDL) into VSMCs by inhibiting the expression of lectin-like oxidized LDL receptor-1 [54]. It is worth noting that lactate dehydrogenase A is essential for the proliferation and migration of VSMCs, and serves as a potential therapeutic target to prevent vessel lumen constriction during the process of atherosclerosis and restenosis [55]. miR638 plays a pivotal role in regulating the PDGF-BB-induced proliferation and migration of human VSMCs via targeting lactate dehydrogenase A [41,56]. Additionally, miR149-5p is demonstrated to decrease PDGF-induced VSMC proliferation, invasion, and migration though the inhibition of its target gene histone deacetylase (HDAC) 4 [57], and miR365 suppresses VSMC proliferation via targeting cyclin D1 [58]. Furthermore, the overexpression of miR365 blocks cell G1/S transition by targeting cyclin D1 along with a downregulation of the proliferating cell nuclear antigen, thereby suppressing VSMC proliferation (Figure 2b) [59]. It is suggested that miR365 is a potential therapeutic strategy for the treatment of restenosis and atherosclerosis.

Tyrosine3-monooxygenase/tryptophan5-monooxygenase activation protein zeta (Ywhaz) is a positive regulator of the p38 MAPK signaling pathway. In VSMCs, miR451 protects against intimal hyperplasia and PDGF-BB-induced VSMC injury via targeting Ywhaz and the following p38 MAPK signaling pathway [60]. Interestingly, miR212-5p targets Ywhaz and lymphocytic leukemia 2 (DLEU2) that are highly expressed in atherosclerosis and PDGF-BB-induced VSMCs. The overexpression of DLEU2 accelerates PDGF-BB-induced VSMC viability, migration, and invasion through the suppression of miR212-5p, associating with an upregulation of Ywhaz [61]. PDGFs (e.g., PDGF-BB) enhance the interaction between PDGFRβ and inositol-requiring enzyme 1α, thereby activating X-box-binding protein 1 (XBP1) splicing and increasing VSMC migration and proliferation. Notably, the spliced XBP1 triggers VSMC migration through the PI3K/protein kinase B (AKT) signaling pathway and activates VSMC proliferation partially via upregulating the miR1274B-mediated mRNA degradation of calponin h1 [62]. miR145 attenuates intimal hyperplasia in a rabbit model by suppressing VSMC proliferation, maintaining cells in a contractile state [63]. Mechanistically, miR-145 could inhibit VSMC proliferation, migration, and phenotype switching by preventing activation of the PI3K/Akt/mTOR signaling pathway [64]. Additionally, miR34a, miR34b, and miR34c are demonstrated to inhibit the PI3K/AKT signaling pathway, thereby decreasing VSMC proliferation and migration [65,66,67]. Several other miRNAs have been demonstrated to modulate neointimal formation [68,69]. For instance, miR612 inhibits the PDGF-BB-induced migration and invasion of VSMCs via targeting AKT-2, inducing cell cycle arrest at the G1 stage; these data suggest that miR612 may be used as a potential therapeutic candidate for neointimal formation in patients with atherosclerosis [70]. Homeobox B13 (HOXB13) is a master regulator in cell differentiation, proliferation, and migration, particularly in vasculogenesis and vascular remodeling, which are common events during neointimal lesion formation in atherosclerosis and post angioplasty restenosis [71,72]. miR17-5p is demonstrated to have a 7-base perfect binding site and a 5-base imperfect binding site with the 3′UTR region of HOXB13 mRNA. Upon PDGF-BB stimulation, miR17-5p mimics inhibit the proliferation and migration of VSMCs and downregulate the levels of MMP-2 and MMP-9, while antagomiR17-5p shows the opposite effects, suggesting a therapeutic strategy for intimal hyperplasia [73]. Another study finds that the knockdown of miR221 and miR222 in rat carotid arteries suppresses VSMC proliferation and neointimal lesion formation after angioplasty via targeting P27 (Kip1) and P57 (Kip2) [74]. Restoring the expression of miR30a-5p also prevents PDGF-BB-induced VSMC phenotype modulation in vitro and inhibits VSMC proliferation in the arterial walls and neointimal hyperplasia in vivo following carotid injury, suggesting a potential therapeutic strategy for decreasing the progression of intima-hyperplasia-related vascular diseases [75].

Except for atherosclerosis, miRNAs are also involved in CAD and heart failure via regulating the PDGF/PDGFR signaling pathways. For instance, miR654-5p is significantly downregulated in the plasma of patients with CAD and in TNF-α- or PDGF-BB-stimulated VSMCs, suggesting miR654-5p plays a critical role in CAD. Mechanistically, miR654-5p targets A disintegrin and metalloproteinase with thrombospondin motifs-7, suppressing the migration and proliferation of VSMCs [76]. Therefore, miR654-5p might serve as a novel therapeutic target for the treatment of CAD. Furthermore, insulin-like growth factor (IGF) 1 is found to suppress cell proliferation and migration in VSMCs [77]. miR379 is downregulated after PDGF-BB stimulation and in circulating blood that is obtained from patients with atherosclerotic CAD [78]. Notably, the expression of miR379 and IGF-1 is inversely correlated in VSMCs. Recent studies demonstrate that miR379 inhibits the proliferation, invasion, and migration of VSMCs via targeting the 3′UTR of IGF-1 (Figure 2b) [78]. The expression of miR665 is downregulated in cardiomyocytes that are separated from chronic heart failure patients; and this miRNA is involved in the regulation of the expression of cardioprotective cannabinoid receptor CB2 in patients with severe heart failure [79]. In vitro, miR665 inhibits cell proliferation, invasion, and migration in PDGF-BB-induced VSMCs potentially by targeting 3′UTR of fibroblast growth factor (FGF) 9 and myocyte enhancer factor 2D. Moreover, the overexpression of miR665 suppresses the expression of β-catenin, c-myc, and cyclin D1. The mechanisms of action of miR665 is supposed to be associated with its downregulation of the Wnt/β-catenin signaling pathway, which interacts with the TGF-β/Smad3 signaling pathway to promote VSMC proliferation [79]. Moreover, FGF9 is also a target gene of miR182. It has been demonstrated that miR182 inhibits the differentiation, proliferation, and migration of rat-derived VSMCs via suppressing the FGF9/PDGFRβ signaling pathway [80]. Some PDGF signaling pathways that are regulated by miRNAs in VSMCs are shown in Table 1A.

### 3.2. Regulation of PDGF/PDGFR Signaling Pathway by miRNAs in Aortic SMCs

In the arterial wall of atherosclerosis obliterans patients, the expression of PDGFRβ is upregulated, which is negatively corelated with the downregulation of miR29a in these clinical samples. The overexpression of miR29a significantly decreases the expression of PDGFRβ, and the transfection of miR29a inhibitors increases the expression of PDGFRβ in human aortic SMCs, suggesting the negative regulation of miR29a on PDGFRβ expression [81]. Moreover, silent information regulator transcript-1 (SIRT-1) improves VSMC function in atherosclerosis [82], potentially via deacetylating P53, thereby inhibiting the activity of P53 on cell cycle progression and differentiation [83,84]. Notably, miR34c promotes the expression and activity of SIRT-1 through the regulation of PDGFRβ, thereby protecting PDGF-BB-induced human aortic SMCs [85].

Let-7g directly suppresses PDGF-B and decreases the PDGF-BB-induced expression of inflammatory genes, such as IL-8, IL-1β, and granulocyte-macrophage colony-stimulating factor, via inhibiting MEKK1/ERK/KLF4 in human aortic SMCs [53]. miR520c-3p inhibits the PDGF-BB-mediated proliferation and migration of human aortic SMCs by targeting the RelA/p65 NF-κB signaling pathway, providing potential therapeutic strategies in atherosclerosis treatment; and the miR520c-3p agomir also decreases serum TG levels by an unknown mechanism (Figure 2e) [51]. miR503 inhibits PDGF-induced human aortic SMC proliferation via targeting the insulin receptor [86]. Additionally, the knockdown of myocardin in human aortic SMCs triggers autophagy and diminishes the expression of SMC contractile proteins [75]. Recent studies have demonstrated that miRNAs play a vital role in the post-translational orchestration of autophagy-related genes. For instance, miR30a has been reported to inhibit autophagy by directly targeting beclin 1 in various types of cells, including rat aortic VSMCs [87,88]. Some PDGF signaling pathways that are regulated by miRNAs in aortic SMCs are shown in Table 1B.

### 3.3. Regulation of PDGF by miRNAs in Endothelial Cells

Endothelial damage promotes platelet activation and cytokine release, such as PDGF-BB, which promote VSMC dedifferentiation [89,90]. Previous studies have shown that the endothelial infiltration of inflammatory cells upregulates the expression of endothelial cell adhesion molecules, such as intercellular cell adhesion molecule-1 (ICAM-1), leading to the adhesion and chemotaxis of leukocytes, further promoting endothelial damage [91]. For instance, PMP-derived miR223 has been shown to decrease ICAM-1-dependent vascular inflammation via suppressing ICAM-1 in the endothelium, thereby limiting leukocyte adhesion to the endothelium (Figure 2a) [92]. PMP-derived miR223 inhibits the IGF-1 receptor, which promotes advanced glycation-end-product-induced endothelial cell apoptosis [93]. miR-345-3p prevents the apoptosis and inflammation of endothelial cells via inhibiting the TGF-β-activated kinase 1/p38 MAPK/p65 NF-κB signaling pathway (Figure 2a) [94]. Let-7g improves endothelial functions by targeting the TGF-β pathway and SIRT-1 expression, inhibiting the formation of atherosclerotic lesions in apolipoprotein-E-deficient mice fed a high-fat diet [95]. Mechanistically, let-7g decreases the signal transduction of the thrombospondin 1/TGF-β receptor 1/Smad2 signaling pathway, leading to reductions in the expression of adhesion molecules and monocyte adhesion to endothelial cells.

In endothelial cells, the high expression of miR221 blocks migration, proliferation, and angiogenesis through the downregulation of c-Kit [96]. In addition, the miR221 expression is markedly downregulated during erythropoietic differentiation and maturation; and this miRNA is inversely corelated with c-Kit expression, a key regulator of erythropoiesis [97]. miR106b-5p binds to the 3′UTR of angiopoietin 2 to induce the migration and tube formation of human umbilical vein endothelial cells; and human cholesteatoma peri-matrix fibroblasts-derived exosomes transport miR106b-5p to endothelial cells and promote angiogenesis via upregulating the expression of angiopoietin 2 [98]. Furthermore, endothelial cells release miR214-containing exosomes to stimulate angiogenesis via the silencing of the ataxia telangiectasia mutated protein in neighboring target cells [99]. Conversely, miR92, a member of the miR17-92 cluster, is revealed as a negative regulator of angiogenesis by binding to the mRNA of the α5 integrin subunit [100]. However, miR92 is highly expressed in young healthy human endothelial cells compared with senescent endothelial cells that have higher levels of oxidative stress and apoptosis [101]. The overexpression of miR92 enhances the viability of endothelial cells under oxidative stress via targeting the AKT signaling pathway. Additionally, the overexpression of miR214 inhibits PDGF-BB-stimulated Pim-1 expression and SMC migration via modulating the epithelial-mesenchymal transition; therefore, the PDGF/miR214/Pim-1 axis may be a potential target for coronary atherosclerotic heart disease [102]. Some PDGF signaling pathways that are regulated by miRNAs in endothelial cells are shown in Table 1C.

### 3.4. Regulation of PDGF/PDGFR Signaling by miRNAs in Primary PASMCs

**Figure 3:**
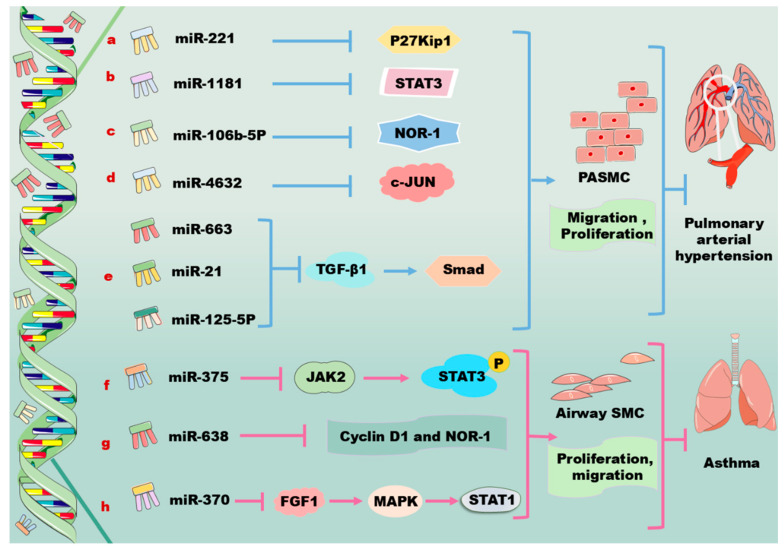
miRNAs target genes that are involved in the PDGF/PDGFR signaling pathways, modulating phenotype switch of PASMCs and airway SMCs, affecting the onset and development of pulmonary arterial hypertension and asthma. miRNAs regulate pulmonary hypertension via activating various molecules including C-Kit, P27Kip1, STAT3, TGF-β1/Smad2/3, PI3K/HDAC4, and NOR-1. miRNAs modulate asthma via targeting the signaling pathways, such as JAK2/STAT3, CyclinD1, NOR-1, and FGF1/MAPK/STAT1. C-Kit: mast/stem cell growth factor receptor kit; FGF1: fibroblast growth factor 1; JAK2: Janus kinase 2; Kip: kinase inhibition protein; MAPK: mitogen-activated protein kinase; NOR-1: neuron-derived orphan receptor 1; PASMC: primary pulmonary artery smooth muscle cell; Smad: drosophila mothers against decapentaplegic protein; STAT: signal transducer and activator of transcription; TGF-β1: transforming growth factor-β1.

miRNAs are involved in the modulation of PASMCs through the PDGF/PDGFR signaling pathway as shown in Figure 3. PDGF induces the expression of miR221, which results in the downregulation of c-kit and p27Kip1 in human primary PASMCs. Notably, the downregulation of c-Kit inhibits the expression of VSMC-specific genes via suppressing myocardin, while the downregulation of p27Kip1 promotes cell proliferation, indicating miR221 exerts distinct effects on PASMCs through the modulation of distinct target genes (Figure 3a) [103]. miR1181 forms a regulatory axis with STAT3, and functions downstream of the PDGF signaling pathway, regulating the proliferation and migration of human PASMCs; this miRNA may have diagnostic and therapeutic potentials for vascular diseases (Figure 3b) [104]. As a member of the NR4A subfamily of nuclear receptors, neuron-derived orphan receptor 1 (NOR-1) is an effector of inflammation, growth factors, lipoproteins, and thrombin; NOR-1 controls the spreading, migration, and proliferation of vascular cells [105,106]. The activity of NOR-1 is upregulated when the cells are affected by an external stimulant. It has been demonstrated that miR106b-5p participates in pulmonary vascular remodeling and decreases excessive cell proliferation and migration via targeting NOR-1 in PDGF-induced PASMCs (Figure 3c) [107,108,109].

Moreover, miR4632 is highly expressed in PASMCs. Functional studies reveal that miR4632 inhibits cell proliferation and promotes cell apoptosis in human PASMCs via suppressing c-JUN; however, this miRNA has no effects on cell contraction and migration. Interestingly, the expression of miR4632 varies in response to different stimuli. For instance, miR4632 is downregulated in PDGF-BB-stimulated human PASMCs; this alteration is associated with its activation of the PDGFR/PI3K/HDAC4 signaling pathway, which leads to histone deacetylation (Figure 3d) [110]. Furthermore, miR663 decreases PDGF-BB-induced PASMC proliferation, migration, and collagen synthesis and prevents pulmonary vascular remodeling and right ventricular hypertrophy in monocrotaline-PAH by targeting the TGF-β1/Smad 2/3 signaling pathway (Figure 3e) [111,112]. It is suggested that the serum levels of miR4632 and miR663 may serve as circulating biomarkers for the diagnosis of PAH [110,111]. Additionally, several other miRNAs, such as miR125-5p and miR21, are also involved in the development of PAH via regulating the TGF-β1/Smad signaling pathway (Figure 3e) [113,114]. It seems that targeting the above-mentioned miRNAs may meet significant clinical benefits in the treatment of PAH through the regulation of the TGF-β1/Smad signaling pathway [111]. Some PDGF signaling pathways that are regulated by miRNAs in PASMCs are shown in Table 1D.

### 3.5. Regulation of PDGF by miRNAs in Airway SMCs

PDGFs induce the proliferation and migration of airway SMCs, contributing to airway hyperresponsiveness and remodeling as shown in Figure 3. Notably, the inhibition of airway SMC proliferation and migration is beneficial for attenuating asthma development [115]. Interestingly, the overexpression of miR375 significantly inhibits the proliferation and migration of airway SMCs that are induced by PDGF via targeting JAK-2, leading to the downregulation of the phosphorylation and the activation of STAT3 (Figure 3f) [115,116]. Consistently, miR375-induced STAT3 inhibition is demonstrated to decrease allergic inflammation, airway remodeling, and airway hyperresponsiveness in mice with asthma via suppressing PDGF-induced airway SMC proliferation [115,117,118]. Moreover, PDGF treatment upregulates the expression of solute carrier family 26 member A2 (SLC26A2) in human airway SMCs, whereas the knockdown of nuclear-enriched abundant transcript 1 (NEAT1) decreases PDGF-induced cell proliferation and migration and the production of inflammatory factors, thereby maintaining cells at a contractile phenotype via the miR9-5p/SLC26A2 axis [119].

miR638, a primate-specific miRNA, is highly expressed in airway SMCs. This miRNA directly targets cyclin D1 and NOR-1, inhibiting airway SMC proliferation and migration in response to PDGF-BB stimulation. In this regard, the overexpression of miR638 in airway SMCs may be a potential therapeutic approach for inhibiting artery airway smooth muscle hyperplasia (Figure 3g) [120]. Phosphatase and tensin homolog deleted on chromosome ten (PTEN) converts phosphatidylinositol-triphosphoric acid into phosphatidylinositol 4,5-bisphosphate and antagonizes the PI3K–AKT–mammalian target of the rapamycin (mTOR) signaling pathway, thus suppressing cell survival, proliferation, and migration [121,122,123]. It has been demonstrated that the overexpression of miR30b-5p facilitates the phosphorylation of PI3K and AKT in PDGF-stimulated airway SMCs by targeting PTEN, upregulating PDGF-induced airway SMC dysfunction [124]. Fibroblast growth factor 1 (FGF1) plays diverse functions in development, wound healing, angiogenesis, neurogenesis, and metabolism [125,126]. miR370 carried by M2 macrophage-derived exosomes alleviates asthma progression by inhibiting the FGF1/MAPK/STAT1 axis in mouse airway SMCs (Figure 3h) [127]. Furthermore, miR370 is reported to target lncRNA X inactivate-specific transcript, thereby reducing cell apoptosis and inflammation injury in acute pneumonia and in PDGF-BB-treated airway SMCs [127,128]. Some PDGF signaling pathways that are regulated by miRNAs in airway SMCs are shown in Table 1E.

**Table 1:** miRNAs are involved in modulation of the PDGF/PDGFR signaling pathways in vascular cells.

A	miRNA	Target	Signaling Pathway	Effects and References
VSMC	miR223 ↑	PDGFRβ ↓	miR223 ↑/PDGFRβ ↓	Platelet-derived miR223 suppresses VSMC differentiation and restores Kawasaki-disease-induced vascular injury [29,30].
	miR145-5p ↑	Smad4 ↓	miR145-5p ↑/Smad4 ↓/PDGF ↓	miR145-5p suppresses PDGF-induced VSMC proliferation and migration [36].
	miR214 ↑	Anti-MIG-6C ↓	miR214 ↑/anti-MIG-6C ↓/TGF-β1 ↓	miR214 promotes profibrotic gene expression [37].
	miR147b ↑	YY1 ↓	miR147b ↑/YY1 ↓/PDGF-BB ↑	Overexpression of miR147b increases the proliferative and migratory abilities of VSMCs [40].
	miR663 ↑	JunB ↓	miR663 ↑/JunB ↓/MMPs ↓	miR663 leads to inhibition of VSMC migration [41].
	miR320 ↑	Neuropilin 1 ↓	miR320 ↑/Neuropilin 1 ↓/PDGF ↓	miR320 inhibits the proliferation and migration of VSMCs in both basal and PDGF-stimulated conditions potentially [42].
	miR340 ↑	VHL ↓	miR340 ↑/VHL ↓	Overexpression of miR340 promotes VSMC proliferation and invasion potentially [43].
	miR125a-5p ↑	EGFR ↓	miR125a-5p ↑/EGFR ↓/PDGF-BB ↑	miR125a-5p suppresses the growth, migration, and invasion of VSMCs [30].
	miR92 ↑	KLF4 ↓	miR92 ↑/KLF4 ↓	miR92 enhances VSMC proliferation and migration [48].
	miR378a-5p ↑	CDK1 ↓	PDGF-BB ↑/miR378a-5p ↑ /CDK1 ↓/p21 ↓	miR378a-5p promotes VSMC proliferation [49].
	miR146b-3p ↑	PI3KCG ↓	PDGF-BB ↓/miR146b-3p ↑/PI3KCG ↓	miR146b-3p reverses the phenotype transition of VSMCs [50].
	miR520c-3p agomiRNA ↑	RelA ↓	miR520c-3p ↑/RelA/p65NF-κB ↓	miR520c-3p agomiRNA decreases atherosclerotic plaque size and collagen content [51].
	Let-7 ↑	Lin-28 HomologB ↓	Let-7 ↑/Lin-28 Homolog B ↓ /TNF-α/IL-6/PDGFR ↑	Let-7 suppresses vascular inflammation mediators, thereby attenuating atherosclerosis as well as diabetes [52].
	Let-7g ↑	MEKK1 ↓, PDGFB ↓	Let-7g ↑/PDGFB ↓, Let-7g ↑/PDGF-BB/MEKK1/ERK/KLF4 ↓	Let-7g can directly suppress PDGF-BB-activated MEKK1/ERK/KLF4 signaling pathway in VSMCs [53].
	miR638 ↑	LDA ↓	miR638 ↑/LDA ↓	miR638 plays a pivotal role in regulating PDGF-BB-induced proliferation and migration of human VSMCs via targeting lactate dehydrogenase A [41,56].
	miR149-5p ↑	HDAC4 ↓	miR149-5p ↑/HDAC4/PDGF ↓	miR149-5p suppresses VSMC proliferation, invasion, and migration [57].
	miR365 ↑	Cyclin D1 ↓	miR365 ↑/cyclin D1 ↓	Overexpression of miR365 suppresses VSMC proliferation [59].
	miR451 ↑	Ywhaz ↓	miR451 ↑/Ywhaz ↓/p38/MAPK ↓	miR451 protects VSMC injury [60].
	miR212-5p ↑	Ywhaz ↓	DLEU2 ↓/miR212-5p ↑/Ywhaz ↓	Overexpression of DLEU2 accelerates PDGF-BB-induced VSMC viability, migration, and invasion [61].
	miR1274b ↑	CNN1 ↓	XBP1 ↑/miR1274B ↑/CNN1 ↓	miR1274B activates VSMC proliferation partially [62].
	miR612 ↑	AKT2 ↓	miR612 ↑/AKT2 ↓/PDGF-BB ↓	miR612 inhibits PDGF-BB-induced migration and invasion of VSMCs [70].
	miR17-5p ↑	MMP-2 ↓, MMP-9 ↓	PDGF-BB ↑/miR17-5p ↑/MMP-2/9 ↓	miR17-5p mimics significantly inhibit the proliferation and migration of VSMCs [73].
	miR30a-5p ↑	PDGF-BB ↓	miR30a-5p ↑/PDGF-BB ↓	miR30a-5p inhibits VSMC proliferation in the arterial walls [75].
	miR379 ↑	IGF-1 ↓	PDGF-BB ↑/miR379 ↑/IGF-1 ↓	miR379 inhibits cell proliferation, invasion, and migration of VSMCs [78].
	miR665 ↑	FGF 9 ↓	miR665 ↑/FGF 9 ↓/PDGF-BB ↑	miR665 inhibits cell proliferation, invasion, and migration in PDGF-BB-induced VSMCs potentially [79].
	miR182 ↑	FGF 9 ↓	miR182 ↑/FGF 9/PDGFRβ ↓	miR182 induces the differentiation, proliferation, and migration of rat-derived VSMCs [80].
**B**	**miRNA**	**Target**	**Signaling Pathway**	**Effects and References**
Aortic SMCs	miR29a ↑	PDGFRβ ↓	miR29a ↑/PDGFRβ ↓	miR29a inhibitors increase the expression of PDGFRβ in human aortic SMCs [81].
	miR34c ↑	PDGFRβ ↓	miR34c ↑/PDGFRβ ↓/SIRT1 ↓	miR34c protects PDGF-BB-induced human aortic SMCs [85].
	Let-7g ↑	MEKK1 ↓	Let-7g ↑/MEKK1/ERK/KLF4 ↓ /PDGFB ↓/PDGF-BB ↓	Let-7g inhibiting MEKK1/ERK/KLF4 in human aortic SMCs [53].
	miR520c-3p ↑	RelA ↓	miR520c-3p ↑/RelA/p65NF-κB ↓	miR520c-3p inhibits PDGF-BB-mediated proliferation and migration of human aortic SMCs [51].
**C**	**miRNA**	**Target**	**Signaling Pathway**	**Effects and References**
Endothelial cells	miR214 ↑	Pim-1 ↓	miR214 ↑/PDGF/Pim-1 ↓	Overexpression of miR214 inhibits SMC migration [102].
**D**	**miRNA**	**Target**	**Signaling Pathway**	**Effects and References**
PASMCs	miR221 ↑	C-kit ↓, P27Kip1 ↓	miR221 ↑/c-kit ↓, miR221 ↑/p27Kip1 ↓	miR221 promotes the proliferation and migration PASMCs [103].
	miR106b-5p ↑	NOR-1 ↓	miR106b-5p ↑/NOR-1 ↓	miR106b-5p decreases excessive cell proliferation and migration in PDGF-induced PASMCs [107,108,109].
	miR4632 ↑	C-JUN ↓	miR4632 ↑/c-JUN ↓	miR4632 promotes cell apoptosis in human PASMCs [110].
	miR663 ↑	TGF-β1 ↓	miR663 ↑/TGF-β1/Smad 2/3 ↓	miR663 decreases PDGF-BB-induced PASMC proliferation, migration, and collagen synthesis and prevents pulmonary vascular remodeling [111,112].
**E**	**miRNA**	**Target**	**Signaling Pathway**	**Effects and References**
Airway SMCs	miR375 ↑	JAK2 ↓	miR375 ↑/JAK2/STAT3 ↓	miR375 suppresses PDGF-induced airway SMC proliferation [115,117,118].
	miR9-5p ↑	SLC26A2 ↓	miR9-5p ↑/SLC26A2 ↓/PDGF ↓	miR9-5p inhibits the PDGF-induced proliferation and production of inflammatory factors in HASMCs [119].
	miR638 ↑	Cyclin D1 ↓, NOR-1 ↓	miR638 ↑/cyclin D1 ↓, miR638 ↑/NOR-1 ↓	miR638 inhibits airway SMC proliferation and migration [120].
	miR30b-5p ↑	PTEN ↓	miR30b-5p ↑/PTEN ↓/PI3K–AKT ↑	Overexpression of miR30b-5p upregulates PDGF-induced airway SMC dysfunction [124].
	miR370 ↑	LncRNA XIST ↓	miR370 ↑/lncRNA XIST ↓	miR370 reduces cell apoptosis and inflammation injury in acute pneumonia and in PDGF-BB-treated airway SMCs [127,128].

**Abbreviations for Table 1**. AKT: protein kinase B; CDK1: cyclin-dependent kinase 1; CNN1: calponin h1; CVD: cardiovascular diseases; CAD: coronary artery disease; DLEU2: lymphocytic leukemia 2; EGFR: epidermal growth factor receptor; ERK: extracellular signal-regulated kinase; FGF9: fibroblast growth factor 9; HDAC4: histone deacetylase 4; ICAM-1: intercellular cell adhesion molecule-1; IGF-1: insulin-like growth factor receptor; IL-6: interleukin-6; JAK2: Janus kinase 2; KLF4: Krüppel-like factor 4; LDA: lactate dehydrogenase A; PAH: pulmonary artery hypertension; NF-ΚB: nuclear factor-kappa B; NEAT1: nuclear enriched abundant transcript 1; MEKK1: mitogen-activated protein kinase kinase 1; MMP: matrix metalloproteinase; MAPK: mitogen-activated protein kinase; MIG-6C: mitogen-inducible gene 6 C; NOR-1: neuron-derived orphan receptor 1; PDGF: platelet-derived growth factor; PI3K: phosphatidylinositol 3-kinase; PI3KCG: phosphoinositide-3 kinase catalytic subunit-gamma; P21: kinase inhibitor cdkn1a; PTEN: phosphatase and tensin homolog deleted on chromosome ten; RelA: reticuloendotheliosis viral oncogene homolog A; SIRT1: sirtuin 1; SLC26A2: solute carrier family 26 member a2; Smad: mothers against decapentaplegic homolog; SMC: smooth muscle cell; TGF: transforming growth factor; TG: triglycerides; TNF-α: tumor necrosis factor-α; VSMC: vascular smooth muscle cell; VHL: von Hippel–Lindau; XIST: x-inactive specific transcript; XBP1: x-box–binding protein 1; Ywhaz: tyrosine3-monooxygenase/tryptophan5-monooxygenase activation protein zeta; YY1: Ying Yang 1.

## 4. lncRNAs in Regulation of PDGF/PDGFR Signaling Pathway in Vascular Cells

**Figure 4:**
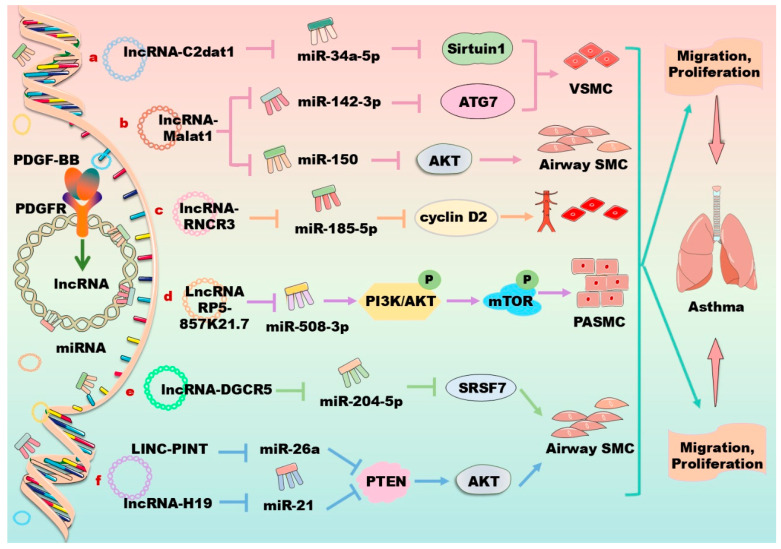
lncRNAs regulate different pathways via sponging miRNAs to modulate cell proliferation and migration as well as inflammation, thereby affecting the occurrence and development of atherosclerosis, asthma, and liver fibrosis. AKT: protein kinase B; AMPK: AMP-activated protein kinase; ATG7: autophagy-related 7; PI3K: phosphatidylinositol 3-kinase; PTEN: phosphatase and tensin homolog deleted on chromosome ten.

In vascular cells, lncRNAs have been demonstrated to modulate cytokine secretion, neovascularization, autophagy, and cellular phenotypic transformation. Mechanistically, lncRNAs act as competing endogenous RNAs for miRNA to modulate the expression of target mRNAs [129]. Therefore, lncRNAs play key roles in the pathogenesis and myocardial regeneration of various CVDs. For instance, some lncRNAs have obvious anti-inflammatory functions, playing significant roles in anti-atherosclerosis, endothelial protection, and so forth. The cardiovascular protective effects of lncRNAs have become a research hotspot in recent years. Moreover, lncRNAs can regulate cell proliferation, migration, and apoptosis in the airway system, exhibiting potential roles for the treatment of asthma. Notably, accumulating evidence has demonstrated that a series of lncRNAs are involved in the regulation of the PDGF/PDGFR signaling pathway in vascular cells as listed in Table 2 and Figure 4.

In human VSMCs, PDGF upregulates the expressions of lncRNAs such as CAMK2D-associated transcript 1 (C2dat1), metastasis-associated lung adenocarcinoma transcript 1 (Malat1), HIX003209, LINC00472, and colorectal neoplasia differentially expressed (CRNDE), and downregulates the expression of other lncRNAs, such as GAS5, HLA complex group 18 (HCG18), and phosphatidylethanolamine binding protein 1 pseudogene 2 (PEBP1P2), thereby modulating cell proliferation, migration, and phenotypic switching [130,131,132,133,134,135,136,137]. For example, PDGF-BB induces the expression of lncRNA C2dat1, which suppresses the expression of miR34a-5p and increases the expression of sirtuin 1, promoting the proliferation and migration of VSMCs through the C2dat1/miR34a/sirtuin 1 axis (Figure 4a) [130]. HIX003209 suppresses miR6089 expression to promote VSMC proliferation and migration and the secretion of inflammatory mediators [131]. The Malat1 overexpression enhances the expression of autophagy-related 7 (ATG7) by competitively sponging miR142-3p, activating the proliferation and migration of VSMCs through enhanced autophagy (Figure 4b) [132]. On the contrary, the knockdown of CRNDE, which is upregulated by PDGF-BB in VSMCs, can abrogate the proliferation and migration of VSMCs [133]. It has been found that lncRNA CRNDE knockdown significantly inhibits the proliferation and migration of VSMCs stimulated by PDGF-BB, suggesting a new target for the diagnosis and treatment of vascular restenosis [133]. LncRNA HCG18 inhibits the proliferation and induces the apoptosis of VSMCs by directly binding with the fused-in sarcoma [134]. Furthermore, the overexpression of KCNQ1 opposite strand/antisense transcript 1 (KCNQ1OT1) suppresses PDFG-BB-induced VSMC proliferation and inflammation by sponging miR221 to upregulate the expression of the inhibitor of NF-κB during intimal hyperplasia in mice [135]. In primary murine VSMCs, PDGF decreases the expression of lncRNA-antisense non-coding RNA in the INK4 locus (lncRNA-ANRIL) to induce the phenotypic switching of VSMCs, and accelerates atherosclerosis development by decreasing the phosphorylation of AMP-activated protein kinase [136]. Furthermore, forkhead box O4 (FOXO4) promotes VSMC migration and dedifferentiation, thereby exacerbating the progression of atherosclerosis. XR007793 negatively regulates the expression of miR23b, enhancing the expression of FOXO4 to aggravate the dysfunction of VSMCs [137].

Vascular aging occurs primarily in the inner and medial layers of the vessel wall, leading to major adverse cardiovascular events including restenosis, atherosclerosis, vascular calcification, and pulmonary hypertension, influencing the onset and development of various CVDs [138]. LncRNAs contribute to vascular aging by acting on chromatin modifications, the cis regulation of target genes, and the post-transcriptional regulation of mRNA processing. It has been demonstrated that lncRNAs respond to extracellular matrix stiffening and regulate VSMC cellular functions. For instance, MALA T1 is a positive regulator of VSMC proliferation and migration in response to extracellular matrix stiffness; the reduction in MALA T1 reduces stiffness-induced proliferation and migration, suggesting a homeostatic negative feedback mechanism [139]. VSMC-enriched lncRNA cardiac mesoderm enhancer-associated non-coding RNA (CARMN) is dynamically regulated along with the progression of atherosclerosis. The knockdown of CARMN significantly reduces atherosclerotic lesion formation by 38% and suppresses VSMC proliferation by 45% without affecting apoptosis in LDL-receptor-deficient mice. Mechanistically, nuclear-localized CARMN interacts with the serum response factor through a specific 600–1197 nucleotide domain [140]. Another study demonstrates that CARMN physically binds to the key transcriptional cofactor myocardin, facilitating its activity and thereby maintaining the contractile phenotype of VSMCs [141]. The LncRNA JPX expression is upregulated in senescent VSMCs and atherosclerotic arteries, and the JPX-enriched chromatin microenvironment mediates VSMC senescence and promotes atherosclerosis. Mechanistically, JPX integrates p65 and bromodomain-containing protein 4 to form a chromatin remodeling complex, activating senescence-associated secretory phenotype gene transcription and promoting cellular senescence. These findings suggest that JPX is a potential therapeutic target for the treatment of age-related atherosclerosis [142]. Additionally, long non-coding RNA differentiation antagonizing non-protein coding RNA (DANCR) is significantly increased in the blood samples of patients with atherosclerosis and oxidized LDL-treated VSMCs. DANCR downregulation obviously increases the viability and reduces the apoptosis of oxidized LDL-treated VSMCs, and miR-214-5p may be a target of this lncRNA [143].

In PDGF-BB-induced human aortic SMCs, the lncRNA small nucleolar RNA host gene 16 (SNHG16) enhances cell proliferation and migration via sponging miR205, thereby increasing the expression of Smad2 [144]. It has been demonstrated that lncRNA hypoxia-inducible factor 1 alpha-antisense RNA 2 (HIF1A-AS2) plays a significant role in aortic dissection. The downregulation of HIF1A-AS2 inhibits the proliferation and migration of aortic SMCs, and promotes the phenotypic transformation of SMCs through the miR33b/high-mobility group AT-Hook 2 axis [145]. Moreover, PDGF-BB stimulation upregulates the expression of lncRNA H19 and PVT1 that sponges miR193b-3p and miR27b-3p, respectively, to induce cell proliferation, migration, and phenotypic differentiation in human aortic SMCs [146,147]. The lncRNA MIAT is a novel regulator of cellular processes in advanced atherosclerosis. This lncRNA controls the SMC proliferation, apoptosis, and phenotypic transition, and the proinflammatory properties of macrophages. MIAT is also involved in the SMC phenotypic transition to proinflammatory macrophage-like cells through binding to the promoter region of KLF4 and enhancing its transcription [148]. In human PASMCs and dermal fibroblasts, PDGF stimulation decreases the expression of lncRNA OTUD6B antisense RNA 1 (OTUD6B-AS1); and silencing this lncRNA decreases cell proliferation and apoptosis by targeting cyclin D1 [149]. In endothelial cells, PDGF-BB induces the expression of lncRNA HOTTIP and RNCR3, which increases proliferation, migration, and inflammatory cytokine secretion via activating the Wnt/β-catenin signaling pathway and miR185-5p/cyclin D2 axis, respectively (Figure 4c) [150,151]. In COVID-19 patients suffering from CVD, clinical complications are caused by the SARS-CoV-2 infection of endothelial cells through the angiotensin-converting enzyme 2 receptor and cellular transmembrane protease serine 2, leading to the consequent dysfunction of infected vascular cells. Recently, accumulating evidence has revealed an important role of lncRNAs in the above processes, opening new opportunities for the treatment of SARS-CoV-2 infection, particularly in the context of CVD [152].

In human airway SMCs, PDGF-BB stimulation suppresses lncRNA-H19 and increases the expression of lncRNAs such as taurine-upregulated gene 1 (TUG1), Malat1, plasmacytoma variant translocation 1 (PVT1), five prime to Xist (FTX), NEAT1, long intergenic non-protein coding RNA 882 (LINC00882), and lncTCF7, thereby regulating asthma and the phenotype of human airway SMCs [153,154]. For instance, lncRNA Malat1 acts as a competing endogenous RNA for miR150, thereby activating AKT signaling and contributing to PDGF-BB-induced airway SMC proliferation and migration (Figure 4b) [153]. Similarly, lncRNA FTX sponges miR590-5p to upregulate the JAK2 signaling pathway, aggravating asthma [154]. LncRNA TCF7 promotes the growth and migration of airway SMCs in asthma via targeting the translocase of the inner mitochondrial membrane domain-containing 1 (TIMMDC1)/AKT axis [121]. LncRNA RP5-857K21.7 is downregulated in PDGF-BB-induced airway SMCs, and its overexpression suppresses the PI3K/AKT/mTOR pathway through sponging miR508-3, significantly inhibiting PDGF-BB-induced cell proliferation and migration and inducing cell apoptosis, providing a new target for the treatment of asthma (Figure 4d) [155]. LncRNA diGeorge syndrome critical region gene 5 (DGCR5) is upregulated in PDGF-BB-stimulated airway SMCs. The downregulation of DGCR5 reverses the effects of PDGF-BB on the proliferation and migration of airway SMCs. These data reveal that the lncRNA DGCR5/miR204-5p/serine and arginine rich splicing factor 7 (SRSF7) signaling axis plays an important regulatory role in the proliferation and migration of airway SMCs (Figure 4e) [156]. In rat airway SMCs, the upregulated lncRNA TUG1 and GAS5 act as sponges of miR138-5p and miR10a, respectively, and the lncRNA BCYRN1 increases the stability of the transient receptor potential 1 (TRPC1) protein, thereby promoting the proliferation and migration of airway SMCs in asthma [157,158,159]. Additionally, the downregulation of human lncRNA-H19 and rat LINC-PINT are found to enhance the PDGF-BB-stimulated abnormal growth of airway SMCs by targeting the PTEN/AKT axis through sponge miR21 and miR26a-5p, respectively (Figure 4f) [160,161].

Some studies have found that lncRNA LOC102551149 is a competing endogenous RNA targeting miR23a-5p through base pairing. The upregulated expression of miR23a-5p activates the PI3K/AKT/mTOR/Snail pathway to activate hepatic stellate cells and induce hepatic fibrosis, providing new therapeutic targets for the treatment of liver fibrosis [162]. Furthermore, lncRNA HOTAIR enhances PI3K/AKT and ERK signaling through PDGFRβ [163]. In PDGF-BB-treated LX-2 hepatic stellate cells, the silencing of non-coding RNA activated by DNA damage (NORAD) inhibits cell migration and invasion via downregulating the expression of miR495-3p, thereby upregulating the expression of sphingosine-1-phosphate receptor 3 to inhibit liver fibrosis [164]. Some signaling pathways that are regulated by lncRNAs are shown in Table 2.

**Table 2:** LncRNAs are involved in regulation of the PDGF/PDGFR signaling pathways in vascular cells.

Cell Type	LncRNA	Target	Signaling Pathway	Effects and References
VSMC	C2dat1 ↑	miR34a-5p ↓	PDGF-BB ↑/lncRNA C2dat1 ↑/ miR34a-5p ↓/SIRT1 ↑	PDGF-BB promotes proliferation and migration of VSMCs [130].
VSMC	KCNQ1OT1 ↑	miR221 ↓	KCNQ1OT1 ↑/miR221 ↓/IκBα ↑	Overexpression of KCNQ1OT1 suppresses PDFG-BB-induced VSMC proliferation and inflammation [135].
Aortic SMCs	SNHG16 ↑	miR205 ↓	LncRNA SNHG16 ↑/ miR205 ↓/Samd2 ↑	LncRNA SNHG16 enhances human aortic SMCs proliferation and migration [144].
Aortic SMCs	H19 ↑	miR193b-3p ↓	LncRNA H19 ↑/miR193b-3p ↓	miR193b-3p induces cell proliferation, migration, and phenotypic differentiation in human aortic SMCs [146].
Aortic SMCs	PVT1 ↑	miR27b-3p ↓	PVT1 ↑/miR27b-3p ↓	miR27b-3p induces cell proliferation, migration, and phenotypic differentiation in human aortic SMCs [147].
PASMCs	OTUD6B-AS1 ↑	Cyclin D1 ↑	PDGF ↓/lncRNAOTUD6B-AS1 ↑/cyclin D1 ↑	Silencing lncRNA OTUD6B-AS1 decreases cell proliferation and apoptosis [149].
Airway SMCs	RP5-857 K21.7 ↑	miR508-3 ↓	LncRNA RP5-857K21.7 ↑/ miR508-3 ↓/PI3K/AKT/mTOR ↓	LncRNA RP5-857K21.7 inhibits PDGF-BB-induced cell proliferation, migration, and inducing apoptosis [155].
Airway SMCs	H19 ↑	MiR21 ↓	H19 ↑/miR21 ↓/PTEN/AKT ↑	Downregulation of human lncRNA-H19 enhances the PDGF-BB-stimulated abnormal growth of airway SMCs [160].
Airway SMCs	LINC-PINT ↑	PTEN ↑	LINC-PINT ↑/PTEN/AKT ↑	Downregulation of human rat LINC-PINT enhances the PDGF-BB-stimulated abnormal growth of airway SMCs [161].
Endothelial cell	HOTTIP ↑	Wnt/β-catenin ↑	LncRNAHOTTIP ↑/Wnt/β-catenin ↑	LncRNA HOTTIP increases proliferation, migration, and inflammatory cytokine secretion [150].
Endothelial cell	RNCR3 ↑	miR185-5p ↓	RNCR3 ↑/miR185-5p ↓/cyclin D2 ↑	LncRNA RNCR3 promotes proliferation, migration, and inflammatory cytokine secretion [151].
Endothelial cell	HOTAIR ↑	PDGFRβ ↑	LncRNA HOTAIR ↑/ PDGFRβ ↑/PI3K/AKT ↑, ERK ↑	LncRNA HOTAIR inhibits the formation of endothelial cell [163].

**Abbreviations for Table 2**. AKT: protein kinase B; C2dat1: CAMK2D-associated transcript 1; ERK: extracellular signal-regulated kinase; IκBα: inhibitor of NF-κBα; KCNQ1OT1: KCNQ1 opposite strand/antisense transcript 1; LINC-PINT: long intergenic non-protein coding RNA, p53-induced transcript; mTOR: mammalian target of rapamycin; OTUD6B-AS1: OTUD6B antisense RNA 1; PASMC: pulmonary artery smooth muscle cells; PDGF: platelet-derived growth factor; PI3K: phosphatidylinositol 3-kinase; PTEN: phosphatase and tensin homolog deleted on chromosome ten; PVT1: plasmacytoma variant translocation 1; SNHG16: small nucleolar RNA host gene 16; SIRT1: sirtuin 1; SMC: smooth muscle cell; SRSF7: serine and arginine rich splicing factor 7; VSMC: vascular smooth muscle cell.

## 5. CircRNAs in Regulation of PDGF/PDGFR Signaling Pathway in Vascular Cells

**Figure 5:**
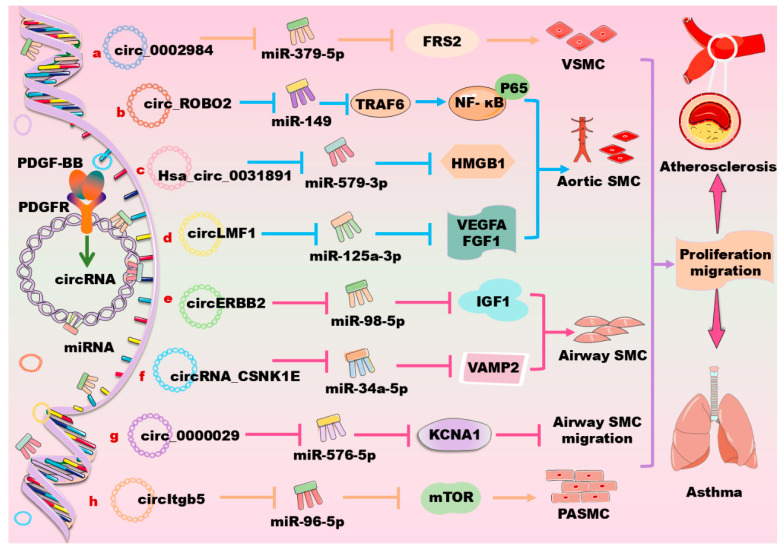
Some circRNAs are found to sponge miRNAs to regulate molecules that are involved in the PDGF/PDGFR signaling pathways, controlling cell proliferation and migration, modulating the onset and development of atherosclerosis and asthma. FGF1: fibroblast growth factor 1; FRS2: fibroblast growth factor receptor substrate 2; HMGB1: high-mobility group box 1; IGF1: insulin-like growth factor 1; KCNA1: voltage-gated potassium channel subfamily A member 1; mTOR: mammalian target of rapamycin; TRAF6: tumor-necrosis-factor-receptor-associated factor 6; VAMP2: vesicle-associated membrane protein 2; VEGFA: vascular endothelial growth factor A.

CircRNAs are a class of single-stranded, closed RNA molecules with unique functions that are ubiquitously expressed in all eukaryotes. Their biogenesis is regulated by specific cis-acting elements and trans-acting factors in humans and animals. CircRNAs mainly exert their biological functions by acting as microRNA sponges, forming R-loops, interacting with RNA-binding proteins, or being translated into polypeptides or proteins in human and animal cells [165]. The targets of circRNAs are widely distributed in distinct vascular cells. Within these cells, circRNAs interact with their target miRNAs to activate multiple signaling pathways, thus controlling the cell cycle, phenotype transformation, angiogenesis, and metastasis. For instance, circ_CHFR has been shown to promote oxidized LDL-induced VSMC proliferation, migration, and inflammation by interacting with miR-214–3p [166]. Therefore, circRNAs play important roles in the occurrence and development of vascular diseases. Moreover, most of the circRNAs have mutual interactions with PDGF/PDGFR signaling. Besides tumors, these circRNAs are expected to become a biomolecular marker for the early diagnosis and/or treatment of CVDs and other vascular-related diseases. The recent findings in these fields are summarized in Table 3 and in Figure 5.

Circ_0002984 promotes PDGF-BB-induced VSMC proliferation, migration, and invasion through the regulation of the miR379-5p/fibroblast growth factor receptor substrate 2 (FRS2) axis, suggesting a novel pathogenesis of atherosclerosis (Figure 5a) [167]. The expression of circ_0006251 is significantly increased in PDGF-BB-induced VSMCs; the downregulation of this circRNA decreases VSMC proliferation and increases cell apoptosis upon PDGF-BB stimulation. Mechanistically, circ_0006251 targets tet methylcytosine dioxygenase 3 (TET3) and protein phosphatase, and Mg^2+^/Mn^2+^-dependent 1B (PPM1B) via sponging miR361-3p, thereby contributing to CAD occurrence [168]. CircHAT1 is downregulated in patients with lower extremity arteriosclerosis obliterans, and this circRNA is demonstrated to inhibit the proliferation, migration, and dedifferentiation of VSMCs upon PDGF-BB induction. Mechanistically, circHAT1 inhibits the proliferation and migration of VSMCs via directly targeting serine and arginine rich splicing factor 1, suggesting a potential therapeutic target for treating lower extremity arteriosclerosis obliterans [169]. Hsa_circ_0000212 (circSFMBT2) is upregulated in neointimal tissue and PDGF-BB-induced VSMCs; this lncRNA regulates VSMC phenotypic modulation via targeting the miR331-3p/HDAC5/angiogenic factor with the G patch and FHA domain axis [170].

Furthermore, PDGF-BB induces the expression of Has_circ_0113656 (circDHCR24), which promotes the proliferation and migration of human aortic SMCs through the circDHCR24/miR149-5p/MMP-9 signaling pathway [171]. Similarly, circSOD2 acts as a sponge for miR206 to promote cell proliferation and migration by upregulating the Notch3/cyclin D1/cyclin-dependent kinase 4/6 signaling pathway in human aortic SMCs [172]. Other mechanisms are also involved in circRNA-mediated cell proliferation and migration in human aortic SMCs. For instance, circ_ROBO2 plays a critical role in the development of CAD via sponging miR149 to activate the TNF receptor-associated factor 6/NF-κB signaling pathway (Figure 5b) [173]. CircPCNX sponges miR1278 to elevate the expression of DNA methyltransferase 1 (DNMT1), promoting PDGF-BB-induced human aortic VSMC proliferation and migration [174]. Hsa_circ_0031891 promotes PDGF-BB-induced human aortic VSMCs proliferation, migration, and dedifferentiation partly via regulating the miR579-3p/high-mobility group box 1 (HMGB1) axis, suggesting a potential therapeutic strategy for atherosclerosis (Figure 5c) [175]. Hsa_circ_0032389 is overexpressed in PDGF-BB-induced human aortic VSMCs, and its downregulation inhibits the human aortic VSMC viability, cell cycle, 5-ethynyl-2′-deoxyuridine positive cell rate, migratory cell number, and wound closure rate; mechanistically, hsa_circ_0032389 enhances PDGF-BB-induced human aortic VSMC proliferation and migration via regulating the miR513a-5p/FRS2 axis [176]. The expression of circ_0004872 is elevated in PDGF-BB-induced human aortic SMCs and carotid plaque tissues; this circRNA promotes PDGF-BB-induced cell proliferation, migration, and dedifferentiation in human aortic SMCs via targeting the miR513a-5p/thioredoxin interacting protein (TXNIP) signaling cascade [177]. Furthermore, circLMF1 is found to regulate the miR125A-3p/VEGF-A and miR125A-3p/FGF1 axis in PDGF-BB-induced human aortic SMCs, thereby accelerating atherosclerosis (Figure 5d) [178].

In asthma, circERBB2 increases the PDGF-BB-induced cell proliferation, migration, and inflammatory response through the modulation of the miR98-5p/IGF1 signaling pathway (Figure 5e) [179]. circ_0002594 acts as a regulator in the airway remodeling during asthma development partly through the modulation of the miR139-5p/tripartite motif (TRIM8) axis [180]. circRNA_CSNK1E promotes the proliferation and migration of airway SMCs through the miR34a-5p/vesicle associated membrane protein 2 (VAMP2) axis upon PDGF-BB stimulation (Figure 5f) [181]. circ_0000029 represses the abnormal migration and growth of airway SMCs by targeting miR576-5p to regulate the expression of KCNA1 (Figure 5g) [182]. circHIPK3 increases the cell proliferation, migration, and apoptosis of airway SMCs during asthma via sponging miR326 and miR375, which target the stromal interaction molecule 1 and MMP-16, respectively [183,184]. Additionally, in PDGF-BB-treated human airway SMCs, the expression of circRNA-4452, circRNA-13360, circRNA-1698, circRNA-8979, and circRNA-14411 is significantly upregulated, whereas the expression of circRNA-3041, circRNA-5780, circRNA-1848, and circRNA-3875 is markedly downregulated, as revealed by a microarray analysis [185].

CircItgb5, a circRNA predominantly localized in the cytoplasm of PASMCs, is significantly upregulated upon PDGF-BB treatment. The knockdown of circItgb5 attenuates monocrotaline-induced pulmonary vascular remodeling and right ventricular hypertrophy in vivo; and this circRNA promotes the transition of PASMCs to a synthetic phenotype in vitro. Mechanistically, circItgb5 sponges miR96-5p to increase the level of mTOR, which ultimately results in an abnormal proliferation of PASMCs [186] (Figure 5h). In retinal vascular endothelial cells that are induced by high glucose and in a diabetic retinopathy mouse model, circFTO (circ_0005941) is found to promote PDGF expression through the downregulation of miRNA-128-3p, thereby upregulating the thioredoxin interacting protein, a downstream gene of miRNA-128-3p [187]. Additionally, circ_0000623 regulates the miR351-5p/transcription factor EB signaling pathway, thereby enhancing lysosomal function and autophagic flux in mouse hepatic stellate cells to improve liver fibrosis [188].

**Table 3:** CircRNAs are involved in modulation of the PDGF/PDGFR signaling pathways in vascular cells.

Cell Type	CircRNA	Target	Signaling Pathway	Effects and References
VSMC	Circ_0002984 ↑	miR379-5p ↓	Circ_0002984 ↑/miR379-5p ↓/FRS2 ↑	Circ_0002984 promotes PDGF-BB-induced VSMC proliferation, migration, and invasion [167].
VSMC	Circ_0006251 ↑	miR361-3p ↓	Circ_0006251 ↑/miR361-3p ↓/TET3 and PPM1B ↑	Circ_0006251 increases VSMC proliferation and decreases cell apoptosis [168].
VSMC	Circ HAT1 ↑	SFRS1 ↓	PDGF ↑/Circ HAT1 ↑/SFRS1 ↓	CircHAT1 inhibits the proliferation and migration of VSMCs [169].
VSMC	CircSFMBT2 ↑	miR331-3p ↓	CircSFMBT2 ↑/miR331-3p ↓/HDAC5 ↑	CircSFMBT2 targets angiogenic factors to regulate phenotypic regulation of VSMC [170].
Aortic SMCs	CircDHCR24 ↑	miR149-5p ↓	PDGF-BB ↑/circDHCR24 ↑ /miR149-5p ↓/MMP-9 ↑	CircDHCR24 promotes proliferation and migration of human aortic SMCs [171].
Aortic SMCs	CircSOD2 ↑	miR206 ↓	CircSOD2 ↑/miR206 ↓Notch3 /cyclinD1/CDK4/6 ↑	CircSOD2 promotes human aortic SMC proliferation and migration [172].
Aortic SMCs	Circ PCNX ↑	miR1278 ↓	Circ PCNX ↑/miR1278 ↓/DNMT1 ↑	CircPCNX promotes PDGF-BB-induced human aortic VSMC proliferation and migration [174].
Aortic SMCs	Hsa_circ_0031891 ↑	miR579-3p ↓	Hsa_circ_0031891 ↑/miR579-3p ↓/HMGB1 ↑	Hsa_circ_0031891 promotes PDGF-BB-induced human aortic VSMC proliferation, migration, and dedifferentiation partly [175].
Aortic SMCs	Hsa_circ_0032389 ↑	miR513a-5p ↓	Hsa_circ_0032389 ↑/miR513a-5p ↓/FRS2 ↑	Hsa_circ_0032389 enhances PDGF-BB-induced human aortic VSMC proliferation and migration [176].
Aortic SMCs	CIrc_0004872 ↑	miR513a-5p ↓	Circ_0004872 ↑/miR513a-5p ↓/TXNIP ↑	Circ_0004872 promotes PDGF-BB-induced cell proliferation, migration, and dedifferentiation in human aortic SMCs [177].
Aortic SMCs	CircLMF1 ↑	miR125A-3p ↓	CircLMF1 ↑/miR125A-3p ↓/VEGFA or FGF1 ↑	CircLMF1 accelerates atherosclerosis [178].
Airway SMCs	CircERBB2 ↑	miR98-5p ↓	CircERBB2 ↑/miR98-5p ↓/IGF-1 ↑	Knockdown of circERBB2 suppresses PDGF-BB-induced cell proliferation, migration, and inflammatory response [179].
Airway SMCs	CircRNA_CSNK1E ↑	miR34a-5p ↓	CircRNA_CSNK1E ↑/miR34a-5p ↓/VAMP2 ↑	CircRNA_CSNK1E promotes proliferation and migration of airway SMCs [181].
PASMCs	CircItgb5 ↑	miR96-5p ↓	CircItgb5 ↑/miR96-5p ↓/mTOR ↑	CircItgb5 results in an abnormal proliferation of PASMCs [186].
Endothelial cells	Circ_0005941 ↑	miR128-3p ↓	Circ_0005941 ↑/miRNA-128-3p ↓/TXNIP ↑	Circ_0005941 promotes PDGF expression [187].

**Abbreviations for Table 3**. CDK: cyclin-dependent kinase; DNMT1: DNA methyltransferase 1; FGF1: fibroblast growth factor; FRS2: fibroblast growth factor receptor substrate 2; HMGB1: high-mobility group box 1; HDAC5: histonedeacetylases; IGF-1: insulin-like growth factor receptor; MMP-9: matrix metalloproteinase-9; mTOR: mammalian target of rapamycin; PASMC: pulmonary artery smooth muscle cell; PDGF: platelet-derived growth factor; PPM1B: protein phosphatase, Mg^2+^/Mn^2+^-dependent 1B; SFRS1: serine and arginine rich splicing factor 1; SMC: smooth muscle cell; TXNIP: thioredoxin interacting protein; TET3: tet methylcytosine dioxygenase 3; VAMP2: vesicle-associated membrane protein 2; VSMC: vascular smooth muscle cell; VEGFA: vascular endothelial growth factors A.

## 6. Concluding Remarks and Future Directions

PDGFs are growth factors that promote mitosis. A variety of cells produce and secrete PDGFs, which induce the cell phenotype switch in distinct vascular cells. SMCs are one of the main components of the arterial wall, and their abnormal proliferation and migration are considered as the critical pathophysiological basis for the occurrence and development of CVDs and respiratory diseases. Therefore, suppressing the proliferation and migration of SMCs may play vital roles for preventing and/or reversing of vascular diseases. Recent studies have demonstrated that PDGF/PDGFR signaling pathways play vital roles in regulating blood vessels, thereby affecting the outcome of CVDs and respiratory diseases. Notably, a series of miRNAs, lncRNAs, and circRNAs are involved in the regulation of PDGF/PDGFR signaling pathways through the regulation of their target genes. Given the fact that the present clinically used drugs cannot completely retard the progression of CVDs and respiratory diseases, these non-coding RNAs provide novel therapeutic strategies for the prevention and treatment of these tricky diseases by the emerging gene therapy.

However, the real clinical application of these therapeutic strategies may have a long way to go due to the following reasons. First, although researchers have made a great amount of progress in this field, the primary theoretical basis and therapeutic effects are obtained using in vitro cellular models and/or in rodents. As the in vitro cellular environments or even the pathological changes of CVDs and respiratory diseases in experimentally induced rodents are significantly different compared with human beings, these data need to be verified in volunteers or in patients in the future. Second, many non-coding RNAs are involved in the modulation of the PDGF/PDGFR signaling pathways and they are demonstrated to be useful for the treatment of various CVDs and respiratory diseases by distinct groups. However, these works have seldomly been repeated by other groups, leading to the potential uncertainty of their effects. Importantly, we do not know which of them are more powerful and valuable due to a lack of comparative studies. Third, due to the complexity and diversity of vascular pathogenesis, the combined effects of the interested strategies via targeting non-coding RNAs and the clinically used drugs need to be investigated in the future. Last but not least, gene therapy may induce side effects. For instance, the marketed miravirsen is developed for the treatment of hepatitis C through the inhibition of miR-122; although miravirsen was shown to be effective, its side effects have not yet been overcome [189].

## Data Availability

Not applicable.
